# Clinical characteristics and follow‑up of Saudi patients with adrenal incidentalomas: single‑center experience

**DOI:** 10.1186/s12902-026-02497-3

**Published:** 2026-08-14

**Authors:** Bader Alharbi, Mohannad Almohaimeed, Eid Aljohani, Mussa Almalki

**Affiliations:** 1 Internal Medicine Department, Second Health Cluster, King Fahad Medical City, Riyadh, Saudi Arabia; 2 Obesity, Endocrine, and Metabolism Center, Second Health Cluster, King Fahad Medical City, Riyadh, Saudi Arabia; 3 College of Medicine, Alfaisal University, Riyadh, Saudi Arabia

**Keywords:** Adrenal incidentaloma, Adenoma, Hormonal evaluation, Saudi Arabia

## Abstract

**Background:**

Adrenal incidentalomas (AIs) are increasingly detected owing to the widespread use of cross-sectional imaging. However, real-world adherence to guideline-recommended evaluation remains variable. This study aimed to determine the prevalence, characteristics, and workup patterns of AIs at a tertiary care center in Saudi Arabia.

**Methods:**

We conducted a retrospective observational study of 250 patients diagnosed with adrenal incidentalomas at King Fahad Medical City between March 2021 and August 2023. Demographic, radiologic, and hormonal data were collected and analyzed.

**Results:**

The mean age was 59.9 ± 13.5 years, with a nearly equal sex distribution. Most lesions were small (57.2% were 1–2 cm) and unilateral (79.6%). Only 20% (*n* = 50) of patients underwent a complete hormonal evaluation. Among these 50 patients, 12.0% (*n* = 6) had a functioning tumor and 88.0% (*n* = 44) were non-functioning; expresedd relative to the entire cohort, functioning tumors corresponded to only 2.4% (*n* = 6), reflecting the impact of the 80% non-evaluation rate on the apparent overall prevelance. Adenomas were the most frequent diagnosis (52.8%), while malignancy was suspected or confirmed in 14.4%. Dedicated adrenal imaging was underutilized, with 94.8% lacking contrast washout studies. Multivariate analysis identified younger age (≤ 60 years), specialty referral, and absence of active malignancy as independent predictors of hormonal evaluation.

**Conclusion:**

This study highlights a substantial gap between clinical guidelines and real-world practice in the evaluation of AIs. Standardized institutional protocols are urgently needed to prevent underdiagnosis of hormonally active or malignant lesions.

**Clinical trial number:**

Not applicable.

## Introduction

An adrenal incidentaloma (AI) is an incidentally discovered adrenal mass measuring ≥ 1 cm, identified on imaging performed for indications unrelated to suspected adrenal disease. With the widespread use of high-resolution cross-sectional imaging, the detection of these lesions has increased substantially over the past two decades. Earlier autopsy and imaging studies reported a prevalence of 0.5–2%; however, contemporary population-based studies now estimate a prevalence of 3–5% in the general population, rising to over 10% among individuals aged > 70 years [1–4].

The clinical relevance of adrenal incidentalomas extends beyond their incidental discovery, as mounting evidence demonstrates an association between these lesions and cardiometabolic morbidity, even in the absence of overt hormonal hypersecretion [5–7]. Accordingly, the detection of an AI mandates a structured diagnostic approach to exclude malignancy and clinically relevant hormone excess.

The recommended diagnostic evaluation includes a detailed clinical assessment, dedicated adrenal imaging, and systematic biochemical screening. On non-contrast computed tomography (CT), benign adrenal adenomas typically present as homogeneous, well-circumscribed lesions with smooth margins, a maximum diameter < 4 cm, and an unenhanced attenuation ≤ 10 Hounsfield units (HU). Features suggestive of malignancy or indeterminate pathology include lesion heterogeneity, irregular margins, attenuation > 10 HU, size ≥ 4 cm, and interval growth [8, 9]. Biochemical evaluation is essential in all patients and includes a 1-mg overnight dexamethasone suppression test (DST) to assess cortisol autonomy, plasma free or 24-hour urinary fractionated metanephrines to exclude pheochromocytoma, and aldosterone-to-renin ratio testing in patients with hypertension and/or hypokalemia [10, 11].

The spectrum of hormonally active AIs includes overt Cushing’s syndrome, mild autonomous cortisol secretion (MACS), primary hyperaldosteronism, and pheochromocytoma. A post-DST serum cortisol ≤ 1.8 µg/dL (50 nmol/L) effectively excludes autonomous cortisol secretion; values > 1.8–5.0 µg/dL are classified as mild autonomous cortisol secretion (MACS) in the absence of overt clinical features of hypercortisolism, whereas levels > 5.0 µg/dL in the presence of compatible clinical features (e.g., proximal myopathy, facial plethora, violaceous striae, easy brusing) are consistent with overt Cushing’s syndrome [10, 12]. This distinction is clinically important, as MACS and overt Cushing’s syndrome carry different prognostic implications and management pathways per the 2023 ESE guidelines. Although the majority of AIs are benign and non-functioning, approximately 20–30% demonstrate some degree of hormonal activity, and a smaller proportion represent malignant lesions. Reported etiologies include adrenocortical adenoma (80–85%), MACS (20–50% of adenomas), pheochromocytoma (1–5%), primary hyperaldosteronism (2–5%), overt Cushing’s syndrome (1–4%), adrenocortical carcinoma (0.4–4%), and adrenal metastases (3–7%) [9, 13].

Large population-based studies have consistently confirmed this epidemiological profile. A UK study evaluating 479,945 imaging examinations reported AIs in 1.2% of scans, with a 14.7% annual increase in detection rates and over 90% occurring in individuals aged > 50 years [14]. A Chinese screening study involving 25,356 participants reported a prevalence of 1.4%, with final diagnoses of non-functioning adenoma (69.3%), cortisol autonomy (18.9%), and primary hyperaldosteronism (11.8%) [15]. A recent meta-analysis of 16,158 patients demonstrated that 27.5% of AIs were hormonally functioning, with MACS being the most prevalent subtype [13].

Data on adrenal incidentalomas from the Middle East, particularly Saudi Arabia, remain limited. A prior single-center Saudi study reported an age-related increase in AI prevalence but emphasized the need for larger, methodologically robust studies to characterize diagnostic practices and outcomes [16].

Given the increasing global burden of adrenal incidentalomas and the paucity of regional data, this study aimed to: (1) determine the prevalence and imaging characteristics of AIs at King Fahad Medical City (KFMC), a tertiary referral center; (2) assess adherence to contemporary diagnostic guidelines for hormonal and radiological evaluation; and (3) estimate the proportion of functioning adrenal lesions within this cohort.

## Materials and methods

### Study design and setting

This was a retrospective observational single-center study conducted at KFMC in Riyadh, Saudi Arabia. The hospital serves as a national referral center for endocrine and metabolic disorders. We reviewed all eligible patients identified through the hospital’s electronic medical records and radiology databases from March 2021 to August 2023.

### Study population

Eligible participants included male and female patients aged ≥ 14 years with an incidentally discovered adrenal mass measuring ≥ 1 cm, detected on imaging performed for indications unrelated to adrenal pathology. Patients were excluded if they had a known history of adrenal disease before the index imaging (including adrenal adenoma, adrenocortical carcinoma, pheochromocytoma, congenital adrenal hyperplasia), known adrenal metastases at presentation, adrenal lesion < 1.0 cm, or age < 14 years. The age threshold of ≥ 14 years was selected to correspond with the institutional threshold for adult endocrine and internal medicine services at KFMC, consistent with regional transition-of-care policies. Adrenal incidentalomas in patients younger than 14 years represent a clinically and diagnostically distinct entity with a different differential diagnosis encompassing pediatric adrenocortical tumors and neuroblastoma and are managed by pediatric endocrinology services at our institution. No patients younger than 14 years were identified in our electronic records during the study period.

### Definitions and diagnostic criteria

AI was defined as an adrenal mass detected incidentally in the absence of clinical suspicion or biochemical evidence of hormone excess at the time of imaging. Non-functioning adenoma was defined as an AI with normal hormonal evaluation and absence of clinical features of hormone excess. MACS was defined as a post-DST serum cortisol > 1.8 µg/dL (50 nmol/L) without overt Cushingoid features. Overt Cushing’s syndrome required compatible clinical features and abnormal results on at least two standard dynamic tests.

Primary hyperaldosteronism was diagnosed in patients with hypertension and/or hypokalemia with elevated aldosterone, suppressed renin, and confirmatory testing when available. Pheochromocytoma was diagnosed based on elevated plasma or urinary fractionated metanephrines with supportive imaging and/or histopathology. Confirmed adrenocrotical carcinoma (*n* = 2) were diagnosed by histopathological examination following surgical rescetion in both cases. Adrenal metastases (*n* = 21) were diagnosed histopathologically in a minority of cases; the majority were classified on the basis of characteristic imaging features (attenuation > 10 HU, heterongenous morphology, irregular margins, and/or interval growth) in patients with a known, active extra-adrenal malignancy, consistent with standard oncologic practice in which adrenal biopsy is reserved for cases in which tissue confirmation would alter management or no known primary malignancy is present. Pheochromocytoma was biochemically excluded prior to any consideration of biopsy. Follow up for non-biopsied metastatic lesions was conducted through each patient’s existing oncologic surveillance imaging rather than a dedicated adreanl-specific protocol. On non-contrast CT, benign imaging features were defined as homogeneous lesions with smooth margins, diameter < 4 cm, and attenuation < 10 HU. Lesions ≥ 4 cm, attenuation > 10 HU, heterogeneous appearance, irregular margins, or evidence of invasion were classified as indeterminate or suspicious. In cases where complete biochemical evaluation was unavailable, final diagnoses were assigned based on the most granular radiological and clinical data accessible in the electronic medical record. Lesions meeting all radiological criteria for probable adenoma (unenhanced CT ≤ 10 HU, homogeneous appearance, smooth margins, diameter < 4 cm, and stable on available follow-up imaging) were classified as adenomas. Lesions with radiological features of malignancy or occurring in the context of known systemic malignancy with imaging consistent with metastatic spread were classified accordingly. Lesions that did not meet predefined criteria for any diagnostic category were classified as indeterminate.

### Data collection

Data were extracted from the hospital electronic medical records, radiology information system, and laboratory databases using a standardized data collection form. Collected variables included demographic data, body mass index (BMI), comorbidities such as hypertension, diabetes mellitus, obesity, dyslipidemia, cardiovascular disease, osteoporosis, and history of malignancy, referring specialty, detailed imaging characteristics, hormonal investigations, and final diagnosis.

Hormonal and imaging evaluation protocol.

Patients were evaluated according to prevailing clinical practice at the time of diagnosis. In general, a 1-mg overnight DST was recommended for all patients. Plasma aldosterone and renin were assessed in hypertensive and/or hypokalemic patients, while pheochromocytoma screening was performed based on symptoms, imaging features, and local protocols. Dedicated adrenal CT was the preferred imaging modality, with further imaging performed when required.

### Outcomes

The primary outcome was the prevalence of adrenal incidentalomas among patients undergoing imaging at KFMC. Secondary outcomes included the proportion of patients receiving guideline-recommended hormonal and imaging evaluation, and the prevalence of functioning adrenal lesions.

### Statistical analysis

Descriptive statistics were summarized as numbers and percentages for all categorical variables, while the mean and standard deviation were given for all continuous variables. The association between hormonal investigation and the demographic and clinical characteristics of the patients was conducted using the Chi-square test and independent sample t-test. Based on significant results, a multivariate regression analysis was performed to identify the significant predictors of hormonal investigation, along with their corresponding odds ratios and 95% confidence intervals. A P-value of 0.05 was considered statistically significant. The data were analyzed using the Statistical Package for the Social Sciences (SPSS) version 26 (Armonk, NY: IBM Corp, USA).

## Results

**Fig. 1:**
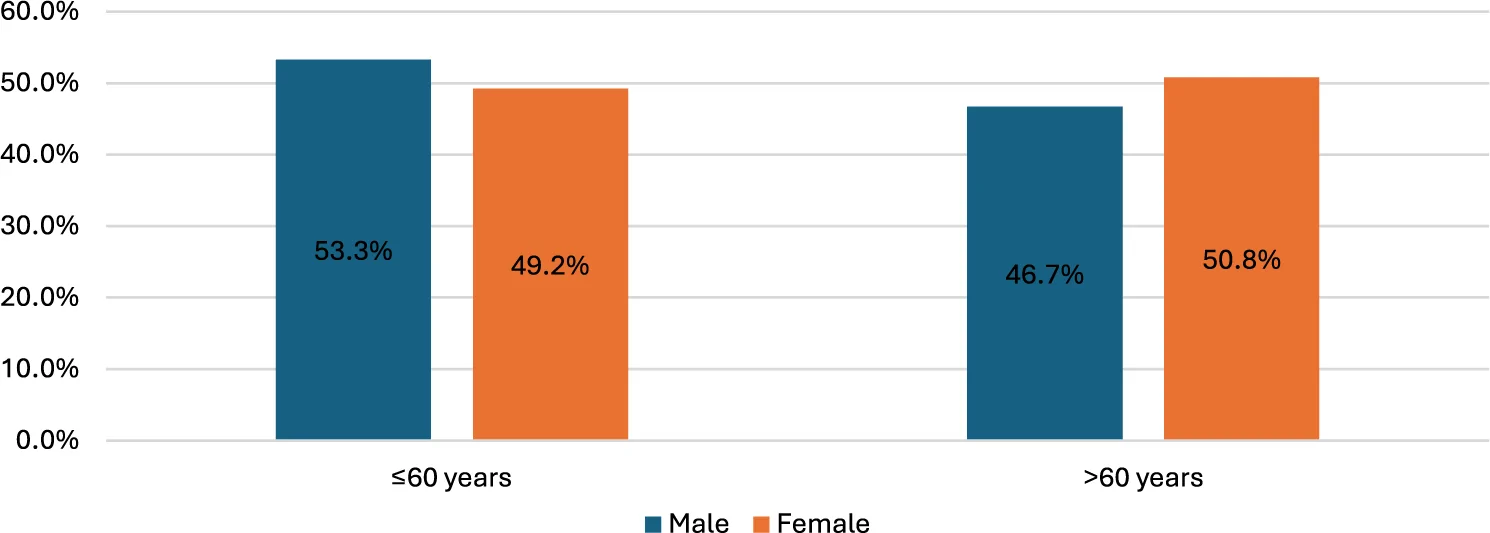
Gender distribution according to age group

This retrospective study included 250 patients with AIs. The cohort had a mean age of 59.9 ± 13.5 years, with a nearly equal distribution between those aged ≤ 60 years (51.2%, *n* = 128) and those aged > 60 years (48.8%, *n* = 122). Overall gender distribution was balanced (48.8% male, 51.2% female); males predominated in the ≤ 60-year group (53.3%), whereas females predominated in the > 60-year group (50.8%) (Fig. 1). Obesity was prevalent, with a mean BMI of 29.2 ± 7.55 kg/m² among patients with available BMI data (*n* = 192). Of these, 43.8% (*n* = 84) of patients had a BMI ≥ 30 kg/m², including 8.9% (*n* = 17) with class III obesity (BMI ≥ 40 kg/m²) (Table 1).

**Table 1:** Baseline demographic and clinical characteristics of patients with adrenal incidentalomas (*N* = 250)

Study variables	*N* (%)
**Age in years (mean ± SD)**	59.9 ± 13.5
• ≤ 60 years	128 (51.2%)
• > 60 years	122 (48.8%)
**Gender**	
• Male	122 (48.8%)
• Female	128 (51.2%)
**BMI kg/m2 (mean ± SD)** ^(*n*=192)^	29.2 ± 7.55
• Non-obese (< 30 kg/m2)	108 (56.3%)
• Obesity Class I (30–34.9 kg/m2)	43 (22.4%)
• Obesity Class II (35–39.9 kg/m2)	24 (12.5%)
• Obesity Class III (≥ 40 kg/m2)	17 (8.9%)

Most adrenal lesions were detected by CT scan (98%, *n* = 245) while MRI (1.2%, *n* = 3) and ultrasound (0.8%, *n* = 2) accounted for a minority. Imaging was performed in inpatient settings in 59.2% (*n* = 148) and outpatient settings in 40.8% (*n* = 102). The referring specialty was undocumented in 82.4% of cases (*n* = 206). Radiology reports frequently lacked detailed morphological characterization, with 82.4% (*n* = 206) reporting no specific morphology. Among reports that included morphological descriptions, the most commonly documented features were hypodense lesion (4%, *n* = 10) and well-defined lesions (3.2%, *n* = 8). Adrenal washout imaging was rarely performed, with washout not done in 94.8% (*n* = 237); among the 5.2% (*n* = 13) with washout data, a washout of 25–49% was the most frequent finding (2.4%, *n* = 6). Most lesions were unilateral (79.6%, *n* = 199) and small with 57.2% (*n* = 143) measuring 1–2 cm and 27.6% (*n* = 69) measuring 2–3 cm (Table 2).

**Table 2:** Imaging characteristics of adrenal incidentalomas

Imaging characteristic	*N* (%)
**Imaging modality**	
• Computed tomography (CT)	245 (98.0%)
• Magnetic resonance imaging (MRI)	3 (1.2%)
• Ultrasound	2 (0.8%)
**Lesion laterality**	
• Unilateral	199 (79.6%)
• Bilateral	51 (20.4%)
**Maximum lesion diameter**	
• 1–2 cm	143 (57.2%)
• 2–3 cm	69 (27.6%)
• 3–4 cm	19 (7.6%)
• > 4 cm	19 (7.6%)
**Dedicated adrenal imaging**	
• Washout study performed	13 (5.2%)
• Washout study not performed	237 (94.8%)
**Morphological description in radiology report**	
• Adequately described	44 (17.6%)
• Not adequately described	206 (82.4%)

**Fig. 2:**
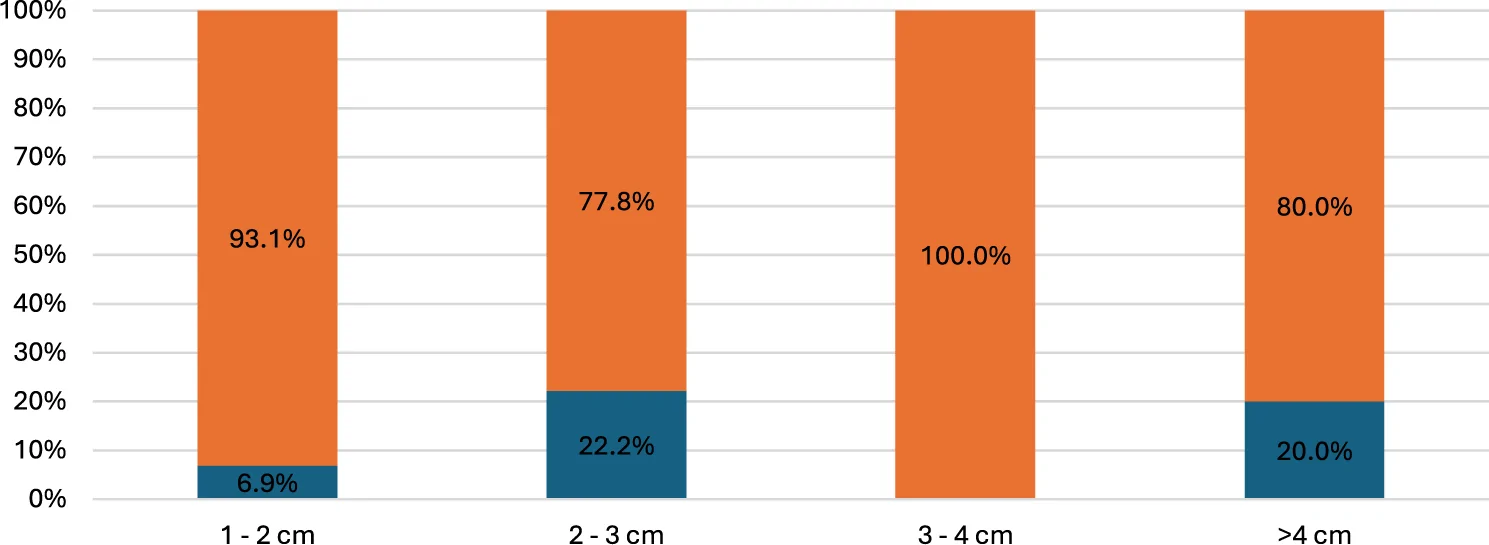
Distribution of adrenal incidentaloma size according to functional status

Hormonal evaluation was performed in only 20% (*n* = 50) of patients, highlighting substantial deviation from guideline-recommended practice. Among the entire cohort, non-functioning lesions accounted for 17.6% (*n* = 44), while functional lesions were confirmed in 2.4% (*n* = 6); however, 80.0% (*n* = 200) were not hormonally investigated. Among the 50 patients who underwent complete hormonal evaluation, non-functioning lesions were identified in 88.0% (*n* = 44) and functioning tumors in 12.0% (*n* = 6). In contrast, when expressed as a proportion of the entire cohort of 250 patients, functioning tumors accounted for only 2.4%, reflecting the impact of the 80% non-evaluation rate on the apparent prevalence of hormonal activity (Table 3). Screening was frequently omitted: metanephrine testing was not performed in 84.0% (*n* = 210), 1-mg DST was not performed in 84.8% (*n* = 212), and aldosterone/renin assessment was not performed in 83.2% (*n* = 208). It should be noted that the 20% (*n* = 50) of patients classified as having undergone hormonal evaluation did not unifolrmly receive the completer recommended biochemical panel. A 1-mg over night DST was performed in 38 patients (15.2% of the cohort; 76% of those evaluated), metanephrines testing in 40 (16.0%), and aldosterone/renin assessment in 42 (16.8%). Testing was frequently selected according to the specific clinical indication prompting evaluation rather than applied uniformly, indicationg that even within the “evaluated” subgroup, biochemical workup up was often partial rather than comprehensive. Notably, no functioning tumors were identified in the 3–4 cm size category, whereas 93.1% of lesions > 4 cm were classified as non-functioning (Fig. 2).

Final pathological or radiological diagnoses revealed that adenomas comprised 52.8% (*n* = 132) of all AIs, followed by metastases (8.4%, *n* = 21) and pheochromocytomas (1.6%, *n* = 4) (Table 4). Confirmed malignancy comprising adrenocortical carcinoma (0.8%, *n* = 2) and adrenal metastases (8.4%, *n* = 21) was identified in 9.2% of patients (*n* = 23). Radiological features suspicious for malignancy without histopathological confirmation were additionally noted in 5.2% (*n* = 13), for a total of 14.4% (*n* = 36) with confirmed or radiologically suspected malignancy. Overall, 21.2% (*n* = 53) lacked a definitive classification.

**Table 3:** Hormonal and biochemical evaluation of patients with adrenal incidentalomas

Study variables	*N* (%)
**Hormonal evaluation**	
• Hormonal evaluation performed	50 (20.0%)
• Hormonal evaluation not performed	200 (80.0%)
Specific hormonal tests performed*	
• Metanephrines testing (plasma and/or urinary)	40 (16.0%)
• Aldosterone/renin assessment	42 (16.8%)
• 1-mg overnight DST	38 (15.2%)
**Hormonal functional status (entire cohort, ** ***N*** ** = 250)**	
• Functioning adrenal incidentaloma	6 (2.4%)
• Non-functioning adrenal incidentaloma	44 (17.6%)
• Not hormonally investigated	200 (80.0%)
**Hormonal functional status (among hormonally evaluated cases**,***n* = 50)**	
• Functioning adrenal incidentaloma	6 (12.0%)
• Non-functioning adrenal incidentaloma	44 (88.0%)

**Table 4:** Final pathological or radiological diagnosis and malignancy status of adrenal incidentalomas

**Diagnosis**	**N (%)**
• Adrenocortical adenoma	132 (52.8%)
• Metastasis	21 (8.4%)
• Pheochromocytoma	4 (1.6%)
• Adrenocortical carcinoma	2 (0.8%)
• Other adrenal pathology	4 (1.6%)
• Not definitively classified	87 (34.8%)
**Malignancy status**	**N (%)**
• Benign	161 (64.4%)
• Malignant	36 (14.4%)
• Not assessed	53 (21.2%)

**Fig. 3:**
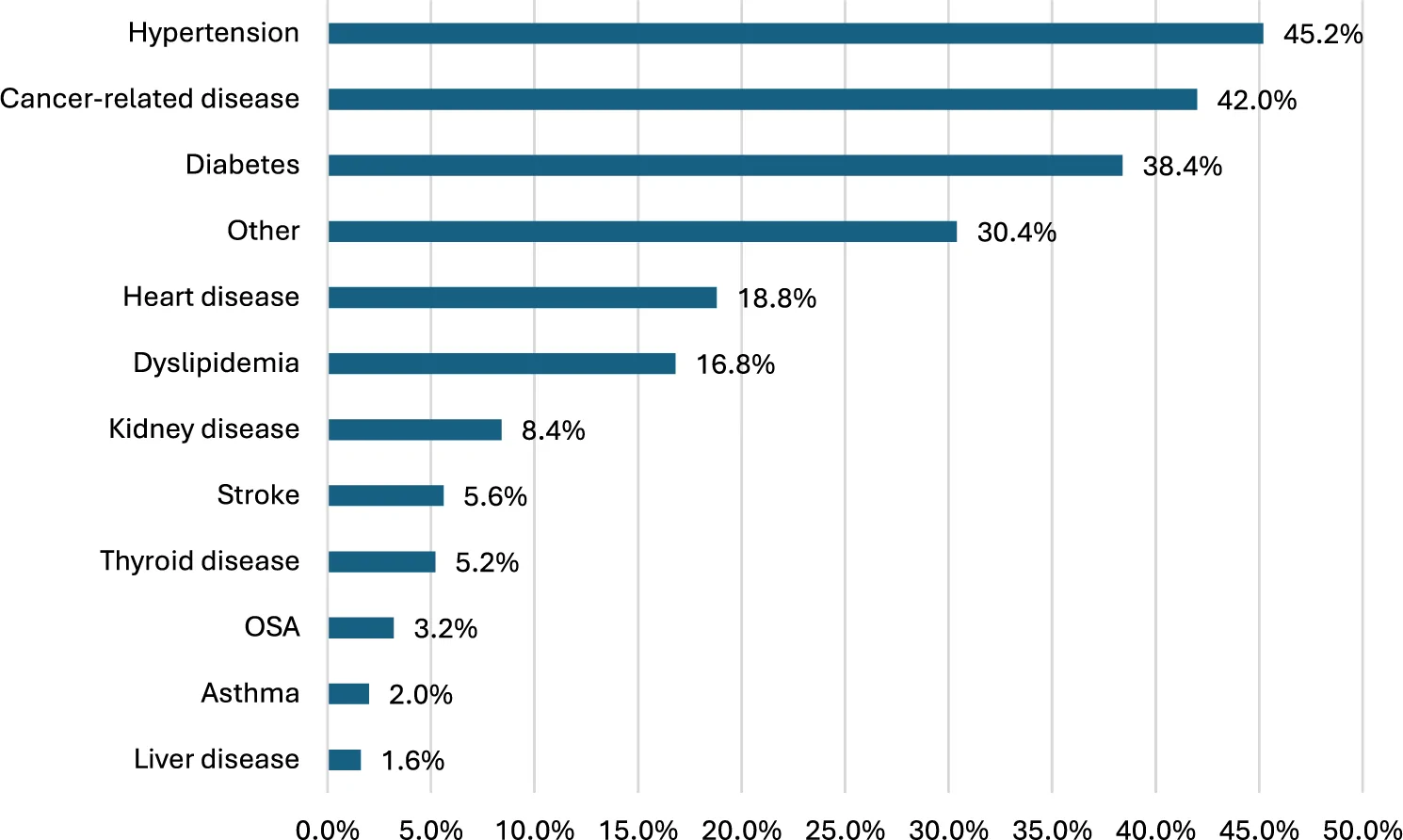
Prevalence of major comorbidities among patients with adrenal incidentaloma

Hypertension (45.2%), cancer (42.0%), and diabetes (38.4%) were the most prevalent comorbidities (Fig. 3). Among patients with hypertension and diabetes, adenoma remained the most common diagnosis, and was slightly more frequent in patients with diabetes (88.6%) than in those with hypertension (84.6%) (Table 5).

**Table 5:** Distribution of adrenal incidentaloma diagnoses according to hypertension and diabetes status

Diagnosis	Hypertension *N* (%)	Diabetes *N* (%)
Adenoma	66 (84.6%)	62 (88.6%)
Metastasis	09 (11.5%)	06 (8.6%)
Pheochromocytoma	02 (2.6%)	01 (1.4%)
Adrenocortical Carcinoma	01 (1.3%)	01 (1.4%)

With respect to lesion size, tumors > 4 cm were more common in patients aged ≤ 60 years (68.4%). Adenomas were most prevalent in the 1–2 cm category (53.1%), and female patients comprised a larger proportion of lesions in the 3–4 cm range (57.9% vs. 42.1% in males). Among patients with lesions > 4 cm, 7 had hypertension and 6 had diabetes. Across size categories, adenoma remained the predominant diagnosis, with the highest proportion observed in the 1–2 cm group (83.5%) (Table 6)

**Table 6:** Clinical characteristics of patients stratified by adrenal incidentaloma size

Characteristics	1–2 cm *N* (%)	2–3 cm *N* (%)	3–4 cm *N* (%)	> 4 cm *N* (%)
Age group				
• ≤ 60 years	73 (51.0%)	32 (46.4%)	10 (52.6%)	13 (68.4%)
• > 60 years	70 (49.0%)	37 (53.6%)	09 (47.4%)	06 (31.6%)
Gender				
• Male	70 (49.0%)	35 (50.7%)	08 (42.1%)	09 (47.4%)
• Female	73 (51.0%)	34 (49.3%)	11 (57.9%)	10 (52.6%)
Hypertension				
• No	77 (53.8%)	34 (49.3%)	14 (73.7%)	12 (63.2%)
• Yes	66 (46.2%)	35 (50.7%)	05 (26.3%)	07 (36.8%)
Diabetes				
• No	91 (63.6%)	33 (47.8%)	17 (89.5%)	13 (68.4%)
• Yes	52 (36.4%)	36 (52.2%)	02 (10.5%)	06 (31.6%)
Diagnosis				
• Not investigated	52 (36.4%)	17 (24.6%)	10 (52.6%)	08 (42.1%)
• Adenoma	76 (53.1%)	41 (59.4%)	07 (36.8%)	08 (42.1%)
• Metastasis	12 (08.4%)	06 (08.7%)	01 (05.3%)	02 (10.5%)
• Pheochromocytoma	01 (0.70%)	03 (04.3%)	0	0
• Adrenocortical Carcinoma	01 (0.70%)	01 (01.4%)	0	0
• Other	01 (0.70%)	01 (01.4%)	01 (05.3%)	01 (05.3%)

Factors associated with hormonal investigation are summarized in Table 7. Hormonal evaluation was significantly more common in patients aged ≤ 60 years (70.0% vs. 30.0%; *p* = 0.003). In contrast, absence of specialty referral was strongly associated with lack of hormonal workup (*p* < 0.001), and patients with cancer-related comorbidity were significantly less likely to undergo hormonal assessment (*p* < 0.001). No statistically significant associations were observed between hormonal investigation and gender, BMI category, lesion laterality, lesion size (≤ 2 vs. > 2 cm), inpatient vs. outpatient setting, or other comorbidities (all *p* > 0.05)

**Table 7:** Association between hormonal investigation and demographic and clinical characteristics

Factor	Hormonal evaluation performed (%) (*n* = 50)	Hormonal evaluation not performed (%) (*n* = 200)	*P*-value ^§^
**Age group**			
• ≤60 years	35 (70.0%)	93 (46.5%)	0.003**
• >60 years	15 (30.0%)	107 (53.5%)	
Gender			
• Male	23 (46.0%)	99 (49.5%)	0.658
• Female	27 (54.0%)	101 (50.5%)	
**Body mass index (kg/m²) ** ***(n = 192)***			
• <30	19 (44.2%)	89 (59.7%)	0.070
• ≥30	24 (55.8%)	60 (40.3%)	
**Adrenal incidentaloma characteristics**			
**Laterality**			
• Unilateral	43 (86.0%)	156 (78.0%)	0.209
• Bilateral	7 (14.0%)	44 (22.0%)	
**Maximum lesion size**			
• 1–2 cm	29 (58.0%)	114 (57.0%)	0.898
• >2 cm	21 (42.0%)	86 (43.0%)	
Clinical comorbidities*			
Hypertension	27 (54.0%)	86 (43.0%)	0.162
Diabetes mellitus	25 (50.0%)	71 (35.5%)	0.059
Cancer-related disease	9 (18.0%)	96 (48.0%)	< 0.001**
**Specialty referral documented**			
• Yes	29 (58.0%)	15 (7.5%)	< 0.001**
• No	21 (42.0%)	185 (92.5%)	
**Imaging setting**			
• Inpatient	26 (52.0%)	122 (61.0%)	0.247
• Outpatient	24 (48.0%)	78 (39.0%)	

* Some patients have more than one comorbidity. ^§^ P-value has been calculated using Chi-square test. ^‡^ P-value has been calculated using independent sample t-test. ** Significant at *p* < 0.05 level

In multivariate logistic regression, three independent predictors of hormonal evaluation were identified: age group, specialty referral, and cancer comorbidity. Patients aged > 60 years had lower odds of hormonal evaluation (AOR = 0.356, 95% CI 0.165–0.766; *p* = 0.008). Specialty referral was strongly associated with hormonal evaluation (AOR = 16.024, 95% CI 6.424–39.972; *p* < 0.001). Presence of a cancer comorbid condition was associated with substantially lower odds of hormonal evaluation (AOR = 0.132, 95% CI 0.049–0.355; *p* < 0.001) (Table 8)

**Table 8:** Multivariate logistic regression analysis for predictors of hormonal investigation

Factor	AOR	95% CI	*P*-value
Age group			
• ≤ 60 years	Ref		
• > 60 years	0.356	0.165–0.766	0.008 **
Patient referral			
• No Referral	Ref		
• Referral	16.024	6.424–39.972	< 0.001 **
Cancer comorbid condition			
• No	Ref		
• Yes	0.132	0.049–0.355	< 0.001 **

(Model adjusted for age, BMI, and hypertension). AOR – Adjusted Odds Ratio; CI – Confidence Interval. ** Significant at *p* < 0.05 level

## Discussion

This single-center, retrospective study of 250 patients with AIs at a tertiary referral center in Saudi Arabia provides valuable real-world insight into contemporary diagnostic practices. Although the demographic and radiographic characteristics were broadly consistent with those reported in international studies, we identified a substantial gap between current guideline recommendations and actual clinical practice, particularly concerning the low rates of hormonal and dedicated adrenal imaging.

The demographic profile of our cohort was characterized by a mean age 59.9 years and a near-equal sex distribution, which is largely consistent with previous epidemiological studies [1, 14]. The finding that nearly half of patients were older than 60 years reinforces the well-established association between AI prevalence and increasing age [1]. Similarly, the predominance of small (1–2 cm), unilateral, and radiologically benign-appearing lesions mirrors findings from the large population-based and referral center cohorts [11, 17]. The high proportion of patients with obesity, hypertension, and diabetes further reflects the metabolic context often associated with AIs [5, 6]. A critical and concerning finding was the notably low rate of guideline-recommended diagnostic evaluation. Only 20% of patients underwent any form of hormonal assessment, and fewer than 6% received dedicated adrenal protocol imaging with washout analysis. It must be acknowledged that this study was not designed to determine the true prevalence of functioning adrenal incidentalomas, but rather to audit real-world adherence to diagnostic guidelines. The 80% rate of non-evaluation reflects precisely the practice gap we sought to quantify. As such, the low detected rate of functioning tumors (2.4%) should not be interpreted as the true epidemiological prevalence within our population, but rather as a consequence of systematic under-investigation. The near-universal absence of dedicated contrast washout imaging (performed in only 5.2% of patients) reflects an additional and distinct dimension of guideline non-adherence. Since washout characteristics are essential for distinguishing adenomas from non-adenomatous lesions in indeterminate cases particularly those with attenuation > 10 HU on non-contrast CT this omission likely contributes to the high proportion of unclassified lesions (21.2%, *n* = 53) observed in our cohort. This substantial under-investigation has direct clinical implications. The proportion of confirmed functioning adenomas in our cohort was only 2.4%, which is markedly lower than the 27.5% reported in a recent meta-analysis of systematically evaluated patients [13] or the 18.9% prevalence of cortisol autonomy reported in a large Chinese screening study [15]. These discrepancies are unlikely to reflect true epidemiological differences and are more plausibly explained by systematic under-evaluation of hormonal function.

Suboptimal adherence to adrenal incidentaloma guidelines is not unique to our institution and has been widely reported across healthcare systems [10, 18, 19]. For instance, a large UK study similarly reported incomplete biochemical evaluation in a significant portion of their cohort [14]. Together, these findings underscore a persistent and international gap between evidence-based recommendations and routine clinical practice. Although the detected prevalence of pheochromocytoma in our cohort (1.6%) falls within the expected range reported in the literature, the fact that over 84% of patients were not screened for catecholamine excess represents a potential safety risk.

Multivariate analysis demonstrated that system-level factors, rather than lesion-related characteristics, were the primary determinants of whether hormonal evaluation was performed.

Younger age and documented referral to a specialty service significantly increased the likelihood of receiving a hormonal evaluation, whereas the presence of an active or prior malignancy was the strongest negative predictor. This finding highlights a common clinical bias, whereby adrenal lesions in oncology patients are frequently presumed to represent metastases, leading to premature diagnostic closure. While this assumption is often correct, it carries the risk of missing a coexisting functional adenoma or a primary adrenal malignancy, either of which could significantly alter patient management.

The overall malignancy rate in our cohort was 14.4%, which is at the higher end of the commonly cited 5–10% range [11, 13]. This is likely attributable to our tertiary care setting, where 42% of the study population had a history of malignancy. Indeed, metastases constituted 8.4% of all final diagnoses. The relatively high prevalence of malignant adrenal lesions is likely attributable to referral bias inherent to a tertiary oncology-referral center. The high rate of imaging during the COVID-19 pandemic, as reported in other regions [20], may also have contributed to the increased detection of incidentalomas in patients with complex comorbidities, including cancer, potentially influencing our cohort’s characteristics. Despite this, benign adrenocortical adenomas remained the most common diagnosis (52.8%), consistent with global data suggesting that 70–80% of AIs are benign and non-functioning [11, 17]. It is likely that the true prevalence of benign adenomas in our cohort is even higher, as more than one-fifth of lesions lacked definitive classification and were predominantly small and radiologically stable.

### Strengths and limitations

The primary strength of this study is its contribution of robust, real-world data from a region where such information is scarce. The relatively large sample size provides a detailed snapshot of current practice patterns and identifies specific areas for quality improvement. However, the findings must be interpreted in light of several limitations. The retrospective design is susceptible to missing data and selection bias. The setting of a tertiary referral center, particularly one with a high volume of oncology patients, likely skewed our population toward more complex cases, inflating the observed malignancy rate and potentially limiting the generalizability of our results to community settings. Finally, the absence of standardized long-term follow-up for all patients precludes assessment of lesion evolution and functional progression in those who were not fully investigated. A further limitation relates to the diagnostic certainty of lesions classified as adrenal metastases: the majority were classified on imaging and clinical grounds rather than histopathology, and no dedicated adrenal-specific follow up protocol was applied, as these patients were followed through pre-existing oncologic surveillance pathways. We also acknowledge that the biochemical thresholds applied in this study, including the post-DST cortisol cut-off used to define mild autonomous cortisol secretion (MACS), reflect current consensus guidelines rather than a definitive, validated gold standard. This area continues to evolve, and lower cutt-off values have been proposed based on emerging cardiometabolic outcome data; further revisions of these thresholds may alter the classificstion of borderline cases in cohorts such as ours.

## Conclusion

Adrenal incidentalomas at our institution are frequently under-investigated despite imaging and demographic characteristics comparable to international cohorts. This practice gap poses a tangible risk of missed functional or malignant disease. Implementation of standardized institutional diagnostic pathways aligned with contemporary guidelines is urgently needed to improve care quality and patient outcomes.

## Data Availability

The datasets used and/or analyzed during the current study are available from the corresponding author on reasonable request.
